# Target organ damage and incident type 2 diabetes mellitus: the Strong Heart Study

**DOI:** 10.1186/s12933-017-0542-6

**Published:** 2017-05-12

**Authors:** Giovanni de Simone, Wenyu Wang, Lyle G. Best, Fawn Yeh, Raffaele Izzo, Costantino Mancusi, Mary J. Roman, Elisa T. Lee, Barbara V. Howard, Richard B. Devereux

**Affiliations:** 1 Hypertension Research Center and Department of Translational Medical Sciences, Federico II University Hospital, via S. Pansini 5, bld 1, 80131 Naples, Italy; 2 Weill Cornell Medicine, New York, NY USA; 3 Center for American Indian Health Research, College of Public Health, University of Oklahoma, Oklahoma City, OK USA; 4 Epidemiology Department, Missouri Breaks Industries Research Inc, Timber Lake, SD USA; 5 MedStar Health Research Institute, Washington, DC USA; 6 Georgetown/Howard Universities Center for Clinical and Translational Studies, Washington, DC USA

**Keywords:** Arterial hypertension, Left ventricular hypertrophy, Left atrial dilatation, Target organ damage, Body composition, Inflammation

## Abstract

**Background:**

Recent analyses in a registry of hypertensive patients suggested that preceding left ventricular (LV) hypertrophy (LVH) and/or carotid atherosclerosis are associated with incident type 2 diabetes, independent of confounders. We assess the relation between prevalent cardio-renal target organ damage (TOD) and subsequent incident type 2 diabetes in a population-based study with high prevalence of obesity.

**Methods:**

We selected 2887 non-diabetic participants from two cohorts of the Strong Heart Study (SHS). Clinical exam, laboratory tests and echocardiograms were performed. Adjudicated TODs were LVH, left atrium (LA) dilatation, and high urine albumin/creatinine ratio (UACR). Multivariable logistic regression models were used to identify variables responsible for the association between initial TODs and incident diabetes at 4-year follow-up (FU).

**Results:**

After 4 years, 297 new cases of diabetes (10%) were identified, 216 of whom exhibited baseline impaired fasting glucose (IFG, 73%, p < 0.0001). Participants developing type 2 diabetes exhibited higher inflammatory markers, fat-free mass and adipose mass and higher prevalence of initial LVH and LA dilatation than those without (both p < 0.04). In multivariable logistic regression, controlling for age, sex, family relatedness, presence of arterial hypertension and IFG, all three indicators of TOD predicted incident diabetes (all p < 0.01). However, the effects of TOD was offset when body fat and inflammatory markers were introduced into the model.

**Conclusions:**

In this population-based study with high prevalence of obesity, TOD precedes clinical appearance of type 2 diabetes and is related to the preceding metabolic status, body composition and inflammatory status.

*Trial registration* Trial registration number: NCT00005134, Name of registry: Strong Heart Study, URL of registry: https://clinicaltrials.gov/ct2/show/NCT00005134, Date of registration: May 25, 2000, Date of enrolment of the first participant to the trial: September 1988

## Background

Similar to arterial hypertension and obesity, type 2 diabetes mellitus is associated with target organ damage (TOD) [1–3]. However, the sequence between major CV risk factor and TOD has been brought into question by evidence that sometimes TOD precedes the clinical appearance of the disease that is thought to cause it. This potential reverse causation has also been postulated for arterial hypertension and for the possibility to optimally control blood pressure by therapy [4, 5].

More recently, analyses performed in the hypertensive population of the Campania Salute Network (CSN) registry demonstrated that LV hypertrophy and/or carotid atherosclerosis are predictors of incident type 2 diabetes [6], independent of a number of confounders, including age, metabolic syndrome, family history of diabetes, duration of hypertension, and number and type of antihypertensive medications. While in the case of hypertension, masked hypertension and non-dipping pattern due to obstructive sleep apnoea, could help explain why LV hypertrophy appears before clinical manifestation of hypertension detected in the doctor’s office, in the case of type 2 diabetes a clear explanation is more difficult. The reported temporal sequence between TOD and clinical presentation of DM was possibly attributed to risk factors both affecting the CV system and leading to type 2 diabetes, to which LV hypertrophy and/or carotid atherosclerosis may be more sensitive, potentially consistent with the postulated possibility of a vascular origin of type 2 diabetes [6].

At present, the generalizability of the findings of the Campagna Salute Network hypertensive registry to unselected populations cohorts has not been evaluated. Accordingly, this analysis has been designed to evaluate the relation between prevalent TOD and subsequent incident type 2 diabetes in the population of the Strong Heart Study (SHS), and to verify whether conditions associated to pre-diabetes help explain the association.

## Methods

### Population

The SHS is a population-based cohort-study of CV risk factors and disease in American Indians originally from 13 communities, as extensively previously described [7, 8], with follow-up examinations of the original SHS cohort approximately 4 and 8 years after the 1st SHS exam in 1989–1992. During the 2nd exam, the SHS cohort members underwent also standard Doppler-echocardiography. The 4th SHS exam, in 2001–2003, enrolled members of large multi-generation families [Strong Heart Family Study (SHFS)], who underwent echocardiography [9, 10].

**Fig. 1:**
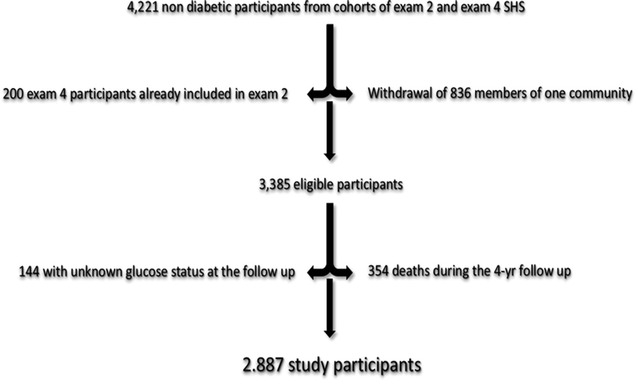
Selection of the study population from the original cohort of non-diabetic participants to the Strong Heart Study and the Strong Heart Family Study

After exclusion of type 2 diabetes participants from the SHS and SHFS cohorts, and withdrawal by one community of consent to use their data, a sub-cohort of 3585 non diabetic participants remained. From the 4th SHS exam, we also excluded 200 participants, who were already included in the present analysis as participants of the 2nd SHS exam. Thus, the study population comprised 3385 participants (1963 or 58% women). Death occurred in 354 participants before the 4-year follow-up. Among the remaining 3031 participants, follow-up data were available in 2887 participants (92%, 1721 women or 60%). Figure 1 displays the steps for selection of the study population.

### Clinical examination, laboratory tests and definitions

Detailed descriptions of the study design and methods of the SHS and SHFS have previously been reported [7–10]. Obesity was classified as BMI ≥30 kg/m^2^. Waist circumference was used as an indicator of central adiposity, using sex-specific cut-points [11]. Arterial hypertension was defined by BP ≥140/90 mmHg or current antihypertensive treatment.

Fat-free mass and adipose body mass were estimated by using an RJL bioelectric impedance meter (model B14101; RJL Equipment Co.), as previously reported [12]. Equations to estimate fat-free mass (FFM) in kg, based on total body water, had been previously validated in the American Indian population [13].

Fasting plasma glucose, lipid profile and other laboratory variables were measured by standard methods, as previously reported [7, 8]. Type 2 diabetes was defined as fasting glucose ≥126 mg/dL or use of antidiabetic treatment [14]. Impaired fasting glucose (IFG) was defined as a fasting glucose >100 mg/dL [11], whereas fasting glucose ≤100 mg/dL was defined as normal fasting glucose (NFG).

Glomerular filtration rate (GFR) was estimated by the simplified Modification of Diet in Renal Disease formula [15]. Urinary albumin excretion was measured on a single spot urine sample and was expressed in relation to grams of urinary creatinine (uAlb/Crea). The ratio uAlb/Crea was also categorized based on the distribution in initially non-diabetic participants in this study, using the 75th percentile of the distribution (i.e. 13 g/mg) as cut point.

### Echocardiographic measures

Echocardiograms were performed using phased-array, commercially available echocardiographs, with M-mode, two-dimensional and Doppler capabilities, and read off line using working stations equipped with frame-grabber to measure on analog stop-frame images, as previously reported in detail [16, 17]. From left ventricular (LV) internal dimension and wall thicknesses, LV mass (LVM) was calculated using an autopsy-validated formula [18] and normalized by height to the allometric power of 2.7 (LVMi) [19]. LV hypertrophy (LVH) was defined using the population specific partition values of 47.2 g/m^2.7^ for both men and women [19]. In the SHS, this cut-point maximized the population risk attributable to LVH, compared to sex-specific approaches. LV geometry was assessed by relative wall thickness (RWT) normalized to an age of 46 years (RWTa), as previously suggested [20]. LV concentricity was defined as RWTa greater than 0.41 [20]. Analysis of reliability of echocardiographic LV mass yielded an intraclass coefficient of 0.93 in this laboratory (p < 0.001) [21].

Left atrial (LA) volume (LAv), in mL was estimated using a recently-validated equation derived from the linear antero-posterior dimension (LAd, in cm), measured in long-axis parasternal view [22]. Thus:

$$LAv = 2.32 \times LAd^{2.07}$$

LAv was normalized for height in meters raised to the second power, based on a recent evidence provided by an European epidemiologic study [23]. From this study, the sex-specific partitions of >19.4 mL/m^2^ for men and >16.6 mL/m^2^ for women were adopted, which provide high sensitivity and specificity for detection of LA dilatation [22].

### Outcome variables

The outcome variable of this study was incident type 2 diabetes defined as fasting glucose ≥126 mg/dL or taking oral antidiabetic medications. Censoring for diabetes was done after 4 years (exams 3 and 5, for the SHS original cohort and for the SHFS, respectively). The markers of TOD evaluated as predictors of incident diabetes were LV hypertrophy, LA dilatation and high uAlb/Crea.

### Statistics

Data were analyzed using IBM-SPSS 23.0 and expressed as mean ± standard deviation or proportion in the Chi square distribution. Indicator variables were included for the Arizona, South/North Dakota and Oklahoma field centers. Because in this population, including members of the SHFS cohort, the level of family relatedness was high, we adjusted analysis for a standard kinship coefficient, based on the level of relatedness within family, as previously reported [24]. Continuous baseline variables were compared between groups with or without incident diabetes, using 2-factor ANCOVA, adjusting for baseline glucose status (i.e. NFG or IFG), and family relatedness. Skewed variables were logarithmically transformed to be analysed with parametric statistics, and are expressed as median and interquartile range. To verify whether the markers of TOD retained their effect on incident diabetes, sequential multivariable logistic regression models were implemented for each TOD marker, to identify confounders (if any) that could help explain its association with incident type 2 diabetes. Because among the variables of interest, we found about 2% of missing values, with no variables exceeding more than 10% of missing values, multiple imputation was adopted, using the SPSS automatic imputing algorithm [25, 26].

## Results

Of the 2887 participants, 1825 presented with normal fasting glucose (NFG, 60% women) and 1062 (57% women) with impaired fasting glucose (IFG).

At the censoring time, 4 years after the initial examination, 297 new cases of type 2 diabetes (10% of the study population) were identified, 216 of whom exhibited baseline impaired fasting glucose (73%, p < 0.0001). At the initial exam, hypertension was present in 30% of the normal fasting glucose (NFG) sub-population and in 48% of participants with IFG (p < 0.0001). Even more marked was the difference in central fat distribution (55% in NGT and 74% in IFG, p < 0.0001).

The following analyses were carried out in the censored subpopulation at follow-up. Incident type 2 diabetes was associated with initial IFG, greater prevalence of obesity, substantial prevalence of central fat distribution, whereas the difference in prevalent arterial hypertension was not significant.

Participants developing type 2 diabetes also exhibited higher blood pressure, heart rate, BMI and waist circumference, fasting insulin and HOMA-R index, and more abnormal lipid profile (lower levels of HDL-cholesterol and higher triglycerides) than those remaining in the non-diabetic range. No between-group difference was detected in age, sex distribution and kidney function. Fibrinogen and PAI-1, the inflammatory markers tested in this analysis, were significantly higher in participants developing type 2 diabetes than in those remaining in the non-diabetic range. Both fat-free mass and adipose mass were also greater in participants developing type 2 diabetes (Table 1).

**Table 1:** Clinical and echocardiographic characteristics of participants remaining non-diabetic and those developing diabetes

	No incident diabetes (n = 2590)	Incident diabetes (n = 297)	p≤*
Age (years)	44 ± 17	47 ± 15	0.101
Sex (% women)	60	61	0.662
Systolic BP (mmHg)	122 ± 17	127 ± 17	0.02
Diastolic BP (mmHg)	75 ± 11	78 ± 11	0.003
Heart rate (bpm)	68 ± 11	70 ± 11	0.02
Body mass index (kg/m^2^)	30 ± 7	35 ± 7	0.0001
Waist circumference (cm)	100 ± 16	113 ± 16	0.0001
GFR_MDRD_ (mL/min/1.73 m^2^)	92 [78–108]	92 [79–109]	0.178^†^
Cholesterol (mg/dL)	187 ± 37	187 ± 39	0.370
HDL-c (mg/dL)	50 ± 15	44 ± 14	0.0001
Triglycerides (mg/dL)	120 [86–171]	147 [107–196]	0.002^†^
Fasting glucose (mg/dL)	96 ± 13	109 ± 25	0.0001
Fasting insulin (mIU/mL)	11 [7–17]	21 [13–31]	0.0001^†^
HOMA-R	2.6 [1.6–4.2]	5.2 [3.4–8.3]	0.0001^†^
Fibrinogen (mg/dL)	347 [304–399]	367 [322–427]	0.01^†^
PAI-1 (mg/dL)	42 [26–65]	58 [38–93]	0.01^†^
Fat-free mass (kg)	53 ± 12	59 ± 14	0.04
Adipose mass (kg)	35 ± 10	40 ± 8	0.0001
LVDD index (cm/m)	3.09 ± 0.25	3.16 ± 0.29	0.008
LV mass index (g/m^2.7^)	38.0 ± 8.8	42.2 ± 10.3	0.0001
Relative wall thickness^a^	0.32 ± 0.05	0.33 ± 0.04	0.006
LAV index (mL/m^2^)^b^	11.4 ± 3.0	13.1 ± 3.0	0.0001
Ejection fraction (%)	63 ± 5	64 ± 5	0.2
Urinary albumin/creatinine	6.55 [4.22–12.08]	8.32 [4.95–16.56]	0.152^†^

*BP* blood pressure, *GFR* glomerular filtration rate, *LV* left ventricle, *LVDD* LV diastolic dimension, *LA* left atrium, *LAV* LA volume

* Adjusted for initial glucose status and kinship coefficients (to account for family relatedness)

† After log transformation. Medians and interquartile range are displayed

^a^Normalized to age 46

^b^Indexed for height to the second power

LV chamber dimension and mass and estimated LA volume were greater in participants developing type 2 diabetes during follow-up, who also exhibited a tendency toward more concentric LV geometry (Table 1). Participants developing type 2 diabetes had a twofold higher prevalence of initial LVH than those remaining with plasma glucose in the non-diabetic range (23 vs. 13%, OR = 2.04 [95% CI = 1.51–2.77], p < 0.0001). Similarly, the prevalence of initial LA dilatation was twofold greater in participants with incident type 2 diabetes (8 vs. 4%, OR = 2.23 [95°CI = 1.39–3.58], p < 0.001). The ratio uAlb/Crea did not exhibit significant difference between the two subpopulations, but participants developing type 2 diabetes were 1.5-fold more likely to have values greater than the 75th percentile of the distribution (32 vs. 23%, OR = 1.55 [1.19–2.01], p < 0.001).

**Fig. 2:**
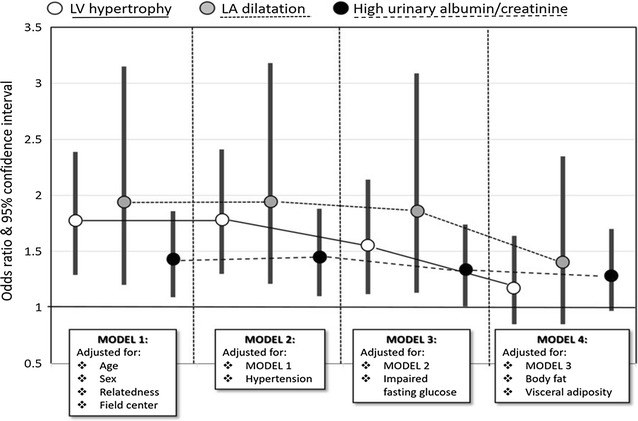
Risk of development of diabetes (expressed as OR and 95% of confidence interval) based on three indicator of target organ damage: LV hypertrophy (*white circles*), LA dilatation (*grey circles*) and high urinary albumin/creatinine excretion (*black circle*). All p value <0.05 except in Model 4

Multivariable logistic regression models were run to determine the possible factors responsible for the association between each indicator of initial TOD and incident type 2 diabetes at 4 years of follow-up. Figure 2 and Table 2 show the odds ratios and corresponding 95% confidence intervals for LVH, LA dilatation and high albumin/creatinine ratio in 4 sequential models forcing incremental sets of potential confounders. In the first models, all three indicators of TOD were associated with incident type 2 diabetes, independently of age, sex and family relatedness (all p < 0.009). This association was not modified when the effect of arterial hypertension was also considered, but was attenuated when initial glucose status entered the model (all p < 0.05). The residual effects of LVH, LA dilatation and high urinary albumin/creatinine excretion were substantially offset when body fat and central fat distribution were introduced into the model (Table 2). Forcing in the last model markers of inflammation did not modify further the impact of TOD on incident type 2 diabetes (ORs were 1.2, p = 0.283 for LVH; 1.42, p = 0.183 for LA dilatation; 1.24, p = 0.141 for high uAlb/Crea).

**Table 2:** Odds of 4-year incident type 2 diabetes mellitus in relation to specific markers of cardio-renal TOD

	LV hypertrophy				LA dilatation				High urinary albumin/creatinine			
	OR	95% CI		p≤	OR	95% CI		p≤	OR	95% CI		p≤
Model 1	1.76	1.29	−2.39	0.0001	1.94	1.20	−3.15	0.007	1.43	1.09	−1.86	0.009
Model 2	1.77	1.30	−2.41	0.0001	1.96	1.21	−3.18	0.007	1.44	1.10	−1.88	0.007
Model 3	1.55	1.12	−2.14	0.008	1.86	1.13	−3.09	0.02	1.32	1.01	−1.74	0.048
Model 4	1.18	0.85	−1.64	0.322	1.41	0.85	−2.35	0.187	1.29	0.97	−1.70	0.077

*Model 1* adjusted for age, sex, family relatedness, field center

*Model 2* adjusted for Model 1 + arterial hypertension

*Model 3* adjusted for Model 2 + impaired fasting glucose

*Model 4* adjusted for Model 3 + Body fat and visceral adiposity

To obtain a phenotypical profile of individuals at high risk of developing type 2 diabetes, in a further analysis, we used all variables of model 4 (as in Fig. 2 and Table 2), also adding inflammatory markers in a stepwise building model. Table 3 displays that the phenotype at risk of type 2 diabetes was a male, with impaired fasting glucose, abnormal body composition (increasing by 6% risk of type 2 diabetes for each kg of adipose mass contributing to his body weight) and high levels of inflammatory markers, with no significant effect of central adiposity.

**Table 3:** Significant predictors of incident diabetes (by backward logistic regression, including all variables displayed in Fig. 2

	b	Wald	p≤	OR	95% Conf. interv.	
Female sex	−0.77	16.30	0.0001	0.47	0.32	−0.67
Impaired fasting glucose	1.45	99.73	0.0001	4.25	3.20	−5.65
Body fat (kg)	0.06	33.84	0.0001	1.06	1.04	−1.09
Fibrinogen (log_10_ [mg/dL])	1.98	6.81	0.009	7.18	1.63	−31.53
PAI-1 (log_10_ [mg/dL])	0.93	14.49	0.0001	2.54	1.57	−4.10

To verify the cofactors that influence the impact of central fat distribution on incident type 2 diabetes, we built a sequential logistic model forcing central fat distribution as the first predictor of incident type 2 diabetes, adjusting for age, sex and relatedness (OR = 3.68 [2.2–5.19], p < 0.0001). In a second step, LVH, impaired fasting glucose and amount of body fat were also forced, and the significant association of central fat distribution with incident type 2 diabetes was attenuated (OR = 1.52 [1.01–2.29], p < 0.05). Finally, inflammatory markers (PAI-1 and fibrinogen) were added in the model with the consequent drop of statistical impact of central fat distribution on probability of incident type 2 diabetes (OR = 1.35 [0.89–2.07], p < 0.155).

## Discussion

In the present study, conducted in an unselected cohort of non-diabetic American Indians, cardiac and renal TOD, generally attributable to type 2 diabetes, preceded the clinical appearance of the disease. This finding confirms, on a population-based scale, findings reported in the observational hypertensive registry of the Campania Salute Network [6]. However, the analysis performed in the SHS cohort at least in part clarifies the possible mechanisms of this apparent reverse-causality association.

### Cardio-renal TOD and incident diabetes

Similar to what reported in the Campania Salute Network [6], in the SHS cohort, cardio-renal TOD was associated with 4-year incident type 2 diabetes, independent of age, sex, family relatedness and, most important, arterial hypertension, but the metabolic phenotype preceding the clinical manifestation of type 2 diabetes could largely explain the temporal association between TOD and incident type 2 diabetes. This metabolic phenotype included insulin resistance, amount of body fat and visceral adiposity. That type 2 diabetes can be predicted by this unfavorable metabolic phenotype is not surprising [27], but it is of note that LV hypertrophy, LA dilatation and, to some extent, proteinuria precede development of the disease.

We have already shown that these abnormalities were present in the adolescent population of the SHFS with IFG [24]. The sequential logistic model we run in the present analysis largely demonstrate that at the basis of the evidence of TOD preceding detection of type 2 diabetes there are metabolic abnormalities with high potential to have impact on the CV system. This observation is well fitting with cross-sectional and longitudinal analyses demonstrating that substantial CV risk attributable to type 2 diabetes is sustained by the coexisting metabolic abnormalities, often clustered in the metabolic syndrome [28–30]. However, the evidence we provide is specific for cardio-renal TOD, and may not be extrapolated to the vascular bed, a district in which the atherosclerotic evolution is more accelerated in type 2 diabetes than in metabolic syndrome without diabetes [31]. This atherosclerotic evolution is also responsible for specific functional alterations in diabetes, such as increased arterial stiffness [32].

### Influence of arterial hypertension

In multivariable logistic regression, arterial hypertension did not influence the relation between cardio-renal TOD and incident type 2 diabetes, though the prevalence of hypertension in this population was high (40%). This finding is fitting with other observations from the SHS and other epidemiological studies [33–35], demonstrating disagreement between evolution of TOD and blood pressure.

It is also of note that the phenotype at risk of type 2 diabetes emerging from our analysis is not substantially different from that associated with 8-year incident hypertension in SHS participants with NFG and optimal blood pressure at the initial observation [36]. In that study, central fat distribution was a key factor also for incident hypertension, and, interestingly, type 2 diabetes detected after 4 years was independently associated with 2.6-fold greater risk of being hypertensive at the censoring time after 8 years follow-up. Considering all together these findings strongly indicate that both type 2 diabetes and, to some extent, hypertension share a common risk phenotype.

### Inflammation

The present analysis also suggests a strong role of central fat distribution-related inflammation in the development of type 2 diabetes [37]. Inflammation was not considered in our analysis on incident hypertension, in which, in fact, central fat distribution was a critical predicting factor [36], as it was in the present logistic model before entering the inflammatory markers. The link between type 2 diabetes and inflammation has been reported in other studies [38].

## Limitations

In the present study, we adopted the simplest possible diagnostic criterion for DM, according to ADA recommendations. However, a further in-depth screening, using 2 h OGTT could have revealed more prevalent diabetes, and could even better refine the selection of the study population. However, though it could be appropriate, the current recommendations do not require further diagnostic tests for subjects classified in the range of impaired fasting glucose and our criterion is more adherent to the present clinical practice.

In the SHS, arterial hypertension has been detected using office and not ambulatory BP monitoring. Because the high prevalence of central obesity and IFG, masked hypertension might not be rare in the SHS participants [39]. Thus, we cannot rule out the possibility that the diagnosis of hypertension at the time of initial examination was underestimated.

In our analysis, sex has been used as a covariate, because the lack of adequate power to run sex-specific analyses. However, sex-differences in the relation between both cardio-renal and vascular TOD and incident diabetes might be relevant. The progression of vascular TOD seems to be more accentuated in women than in men [32, 40], and the prevalence of cardiac TOD is higher in women, especially in the presence of central obesity and abnormal body composition [12, 41], both factors predicting incident diabetes.

## Conclusions

In this population-based study, we demonstrated that cardio-renal TOD generally attributable to type 2 diabetes precedes the clinical detection of the disease, but is substantially related to the metabolic status preceding diabetes including IFG, body fat accumulation and inflammation.
